# Anaphylaxis-Associated Cerebral Infarction: A Case Report

**DOI:** 10.7759/cureus.80887

**Published:** 2025-03-20

**Authors:** Tatsuya Watanabe, Hidetoshi Yamana, Koji Ishigami, Yusuke Tsutsumi, Noriyuki Kato

**Affiliations:** 1 Department of Emergency Medicine, National Hospital Organization (NHO) Mito Medical Center, Ibaraki, JPN; 2 Department of Emergency and Critical Care Medicine, University of Tsukuba Hospital, Tsukuba, JPN; 3 Department of Neurological Surgery, National Hospital Organization (NHO) Mito Medical Center, Ibaraki, JPN

**Keywords:** acute cerebral infarction, anaphylaxis, anti-platelet agents, bee-sting, kounis syndrome, perfusion weighted imaging(pwi), thrombolytic therapy

## Abstract

Anaphylaxis is a severe, potentially life-threatening allergic reaction that primarily affects the respiratory and cardiovascular systems. While neurological complications are rare, cerebral infarction may occur due to anaphylaxis-induced hypoperfusion, endothelial dysfunction, and a hypercoagulable state. The mechanisms underlying this association remain poorly understood, and reports of anaphylaxis-related stroke are scarce.

A 75-year-old man with a history of diabetes mellitus and hypertension developed respiratory distress and loss of consciousness shortly after a bee sting. He was diagnosed with anaphylactic shock and treated with adrenaline, glucagon, and aggressive fluid therapy. Despite hemodynamic stabilization, he developed persistent left-dominant quadriplegia. Magnetic resonance imaging (MRI) revealed acute ischemic stroke confined to the cortical branches of the middle cerebral artery (MCA) bilaterally, with restricted diffusion on diffusion-weighted imaging (DWI) and decreased apparent diffusion coefficient (ADC) values. Magnetic resonance angiography demonstrated a signal void in the right MCA and stenosis of the left MCA. Perfusion-weighted imaging using time-to-maximum maps indicated prolonged transit time in the infarcted regions, which closely corresponded to the DWI lesions, suggesting that most of the hypoperfused tissue had already progressed to irreversible infarction. Given the extensive infarction and high risk of hemorrhagic transformation, single antiplatelet therapy was selected for secondary stroke prevention. While his neurological symptoms partially improved, he remained functionally dependent at discharge.

Cerebral infarction is a rare but serious complication of anaphylaxis, especially in patients with underlying cerebrovascular risk factors. Early recognition of neurological deficits, prompt neuroimaging, and comprehensive hemodynamic stabilization are crucial in preventing irreversible brain ischemia. The underlying pathophysiology is thought to involve multiple mechanisms, including systemic hypotension, mast cell degranulation-induced endothelial dysfunction, transient or persistent cerebral vasoconstriction, and a hypercoagulable state triggered by inflammatory mediators. Considering the etiology of anaphylaxis-associated ischemic stroke is critical for guiding appropriate management strategies. This case highlights the importance of a multidisciplinary approach in managing anaphylaxis-related stroke. Further studies are warranted to clarify the pathophysiological mechanisms and develop tailored therapeutic strategies for this rare but severe condition.

## Introduction

Anaphylaxis is a severe and potentially life-threatening systemic allergic reaction that can rapidly affect multiple organ systems, notably the respiratory, cardiovascular, and gastrointestinal systems [1,2]. Common triggers include food, drugs, insect stings, and other allergens, which initiate a cascade of inflammatory mediator release [3]. These mediators provoke dramatic physiological changes, such as vasodilation, increased vascular permeability, and airway obstruction [2,3]. Prompt administration of adrenaline is the cornerstone of anaphylaxis management, effectively reversing hypotension and mitigating life-threatening symptoms [1]. Despite appropriate treatment, severe anaphylaxis may follow a complicated clinical course characterized by prolonged circulatory instability and multi-organ involvement [4]. Although cardiovascular and respiratory complications are well-documented, neurological complications following anaphylaxis are exceedingly rare and remain poorly understood [5]. Several mechanisms have been proposed to explain anaphylaxis-associated cerebral infarction, including hypoperfusion due to anaphylactic shock, vasospasm triggered by mast cell degranulation, dehydration, and a hypercoagulable state induced by systemic inflammation [3,6,7]. However, the exact pathophysiological pathways remain unclear. Here, we report a rare case of cerebral infarction following an anaphylactic reaction, underscoring the importance of early recognition, timely neuroimaging, and prompt neurological evaluation in patients with persistent or atypical symptoms after anaphylaxis.

## Case presentation

A 75-year-old man with a past medical history of diabetes and hypertension was stung by a bee while at work. Within minutes of the sting, he developed respiratory distress and lost consciousness, prompting an emergency transport to the hospital. Upon arrival, his vital signs were as follows: Glasgow Coma Scale score, E1V1M4; unmeasurable blood pressure, non-palpable radial pulse, and a palpable femoral pulse. Physical examination revealed diffuse erythema and urticarial lesions, primarily on the trunk. His pupils were bilaterally 4 mm and reactive to light, with no obvious conjugate deviation. Intramuscular adrenaline (0.5 mg per dose) was administered every five minutes (a total of six doses, 3 mg in total), along with intravenous glucagon 1 mg every five minutes (a total of 4 mg). Intravenous fluids were rapidly administered, and a continuous intravenous infusion of adrenaline (1 μg/kg/min) was initiated. Following the start of the adrenaline infusion, blood pressure stabilized, and erythema improved. However, his level of consciousness remained depressed (E2V1M4), and he developed left-dominant quadriplegia affecting both the upper and lower extremities. Manual Muscle Testing (MMT) scores were as follows: right upper limb, 2/5; left upper limb, 1/5; right lower limb, 2/5; left lower limb, 1/5. His National Institutes of Health Stroke Scale score was 29. His electrocardiogram showed normal sinus rhythm. Laboratory tests indicated hemoconcentration, with a hematocrit of 58.6%, hemoglobin of 19.0 g/dL, and a red blood cell count of 5.96 ×10⁶/μL. Coagulation studies demonstrated a markedly elevated D-dimer level of 55.5 μg/mL. Arterial blood gas analysis, performed under a reservoir oxygen mask at 10 L/min, revealed lactic acidosis (pH 7.293, base excess −11.1 mmol/L, and lactate 5.4 mmol/L) (Table 1).　

**Table 1:** Laboratory findings on admission The table summarizes the patient’s initial laboratory results, including complete blood count, coagulation studies, serum chemistries, and arterial blood gas analysis. Laboratory abnormalities included elevated hematocrit (58.6%), hemoglobin (19.0 g/dL), and red blood cell count (5.96 ×10⁶/μL), suggesting hemoconcentration. A markedly elevated D–dimer level (55.5 μg/mL) was observed. Arterial blood gas analysis under a reservoir oxygen mask at 10 L/min revealed lactic acidosis (pH 7.293, base excess -11.1 mmol/L, and lactate 5.4 mmol/L).

Laboratory parameter	Patient’s value	Reference range
Complete Blood Count		
Hemoglobin (g/dL)	19.0	13.7–16.8
Red blood cell count (×10^6^/μL)	5.96	4.35–5.55
Hematocrit (%)	58.6	40.7–50.1
Platelet count (×10^3^/μL)	198	158–348
Coagulation Studies		
Prothrombin time–international normalized ratio (INR)	1.04	0.8–1.1
Activated partial thromboplastin time (sec)	33.5	24–39
D-dimer (μg/mL)	55.5	<0.5
Serum Chemistries		
Creatinine (mg/dL)	1.30	0.65–1.07
Aspartate aminotransferase (U/L)	97	13–30
Alanine aminotransferase (U/L)	82	10–42
Lactate dehydrogenase (U/L)	378	124–222
Arterial Blood Gas Analysis (under reservoir oxygen mask at 10 L/min)		
pH	7.293	7.35–7.45
Partial pressure of carbon dioxide (mmHg)	38.7	35–45
Partial pressure of oxygen (mmHg)	403.1	80–100
Bicarbonate (mmol/L)	21.3	20–26
Base excess (mmol/L)	-11.1	-3.0–3.0
Lactate (mmol/L)	5.4	0.5–2.0

**Figure 1:**
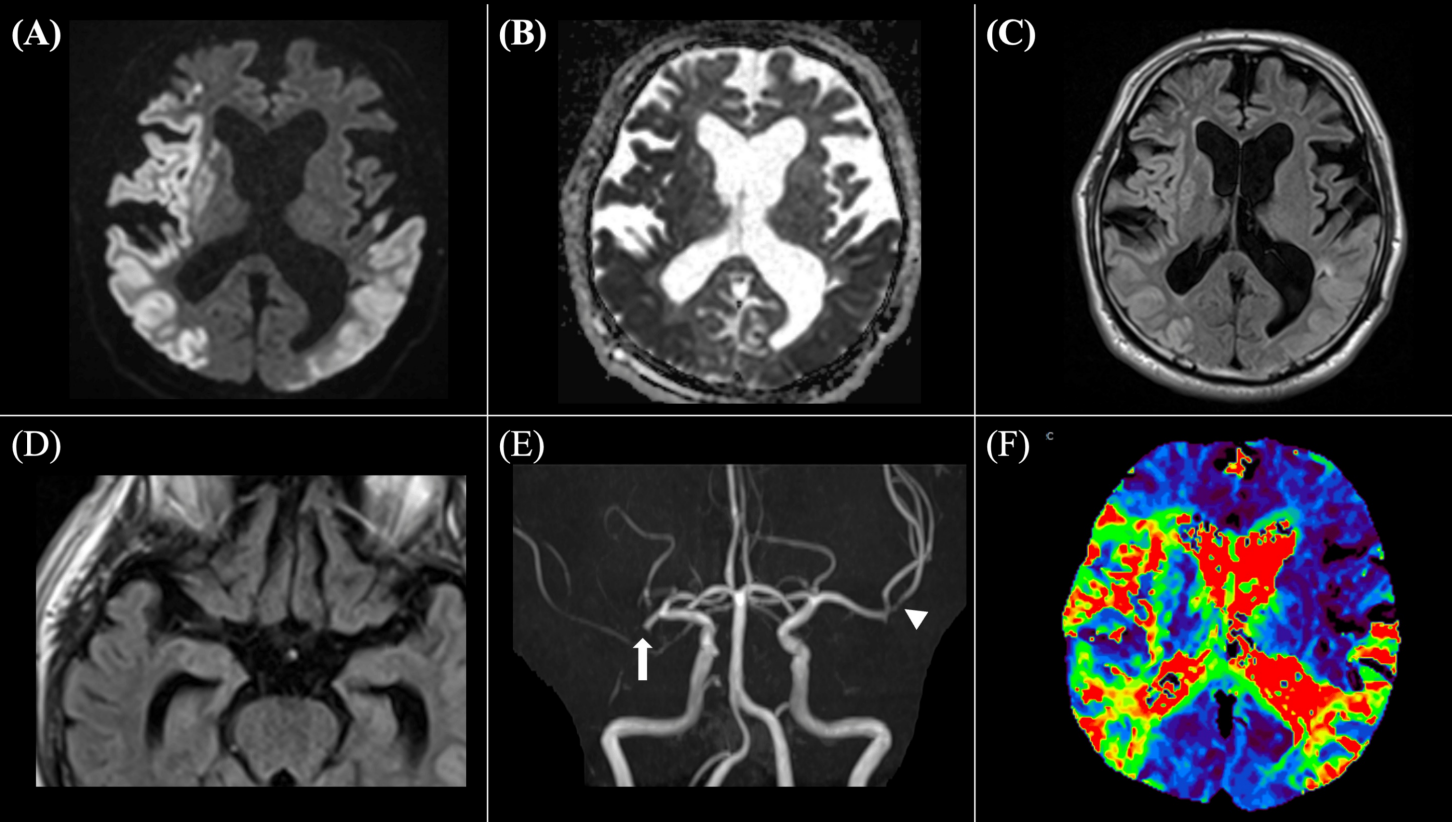
Initial magnetic resonance imaging findings on hospital admission A: diffusion-weighted imaging (DWI) shows acute-phase ischemic changes confined to the gray matter in the upper and middle trunk regions of the right middle cerebral artery (MCA) cortical branch territory and in the lower trunk region of the left MCA cortical branch; B: corresponding apparent diffusion coefficient (ADC) map demonstrates reduced ADC values consistent with infarcted area on DWI; C: fluid-attenuated inversion recovery (FLAIR) imaging reveals increased signal intensity in the infarcted regions seen on DWI, indicating vasogenic edema and evolving infarct; D: FLAIR imaging shows no intra-arterial signals in the bilateral MCAs; E: time-of-flight (TOF) magnetic resonance angiography (MRA) demonstrates a signal void at the proximal M1 segment of the right MCA (arrow) and luminal narrowing of the left MCA (arrowhead); F: perfusion-weighted imaging (PWI) using time-to-max (Tmax) maps shows perfusion deficits corresponding to the infarcted cortical regions seen on DWI, indicating impaired cerebral perfusion. Red, yellow, and green areas represent regions with prolonged transit time (>6s).

Initial magnetic resonance imaging (MRI), performed approximately five hours after anaphylaxis onset due to the need for initial stabilization and logistical constraints, revealed extensive acute-phase ischemic changes confined to the gray matter in the upper and middle trunk regions of the right middle cerebral artery (MCA) cortical branch territory and in the left MCA cortical branch territory, specifically the lower trunk region, on diffusion-weighted imaging (DWI) (Figure 1A). The affected areas exhibited decreased apparent diffusion coefficient (ADC) values (Figure 1B) and high signals on fluid-attenuated inversion recovery (FLAIR) imaging (Figure 1C). Additionally, there was evidence of cytotoxic and vasogenic edema confined to the gray matter, with subcortical low-intensity signals (Figure 1C). No intra-arterial signals were detected on FLAIR imaging, suggesting the absence of embolic occlusion (Figure 1D). Time-of-flight (TOF) imaging revealed a signal void at the proximal M1 segment of the right MCA and stenosis of the left MCA at the M2 segment (inferior trunk) (Figure 1E). Additionally, perfusion-weighted imaging (PWI) using time-to-maximum (Tmax) maps revealed prolonged transit time in the infarcted regions, which closely corresponded to the DWI lesions, suggesting that most of the hypoperfused tissue had already progressed to irreversible infarction (Figure 1F). These neuroimaging findings confirmed acute ischemic stroke and supported a pathophysiology related to anaphylaxis-induced vascular dysfunction rather than embolic sources.

**Figure 2:**
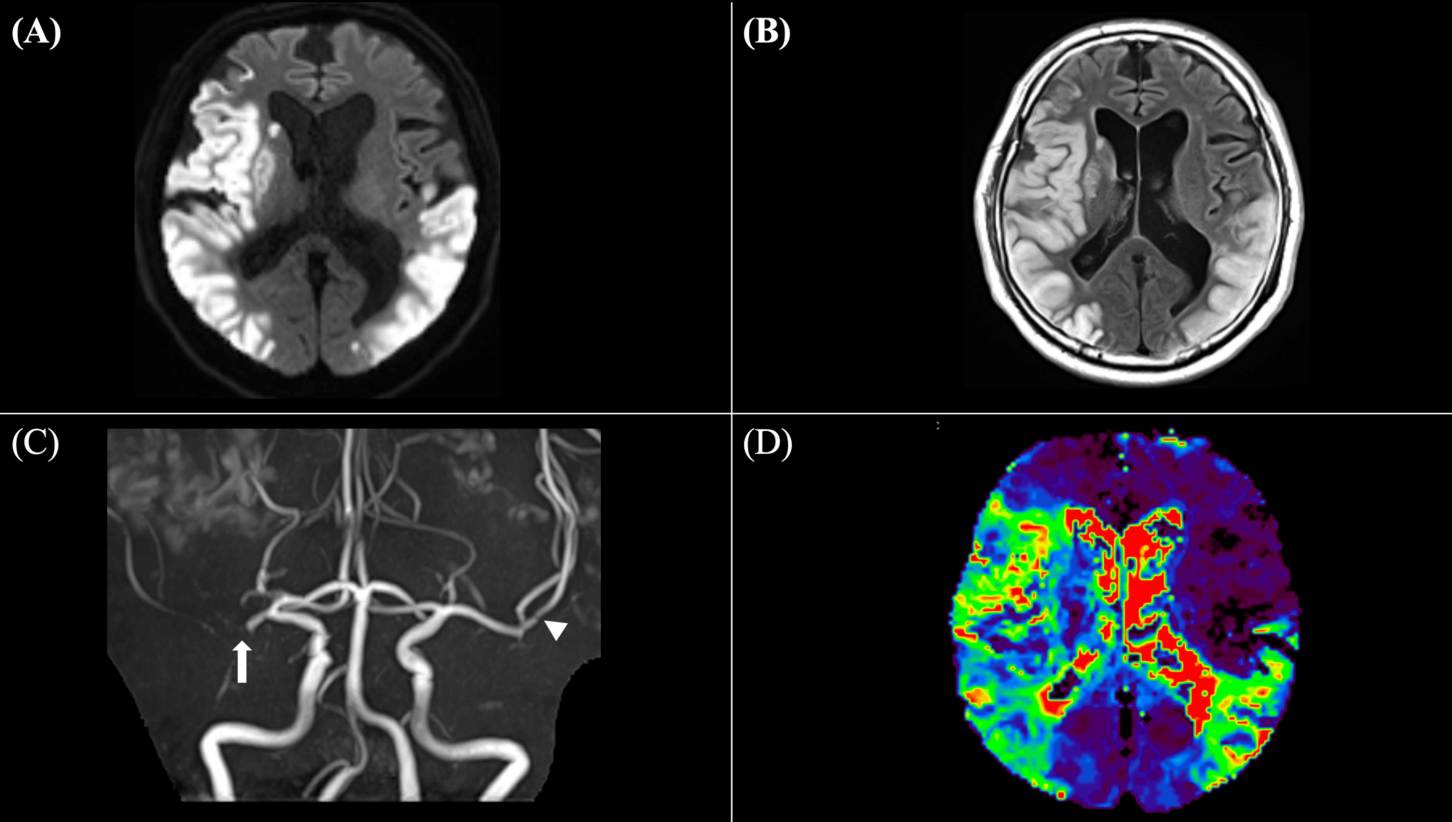
Follow-up MRI findings on hospital day 2 A: diffusion-weighted imaging (DWI) shows no significant enlargement of the infarcted area, suggesting no progression into the subcortical or deep white matter; B: fluid-attenuated inversion recovery (FLAIR) imaging reveals worsening cytotoxic and vasogenic edema in the cortical infarction regions; C: time-of-flight (TOF) magnetic resonance angiography (MRA) demonstrates persistent right middle cerebral artery (MCA) occlusion (arrow) and continued stenosis of the left MCA (arrowhead), without evidence of recanalization; D: perfusion-weighted imaging (PWI) using time-to-max (Tmax) maps shows no significant change in perfusion deficits compared to the initial MRI, indicating persistent cerebral hypoperfusion.

On hospital day 2, a computed tomography (CT) scan confirmed the absence of cerebral hemorrhage, and enteral administration of aspirin 100 mg was initiated as part of secondary stroke prevention. Given the extensive infarction and coagulation abnormalities, the risk of hemorrhagic transformation was considered high, leading to the decision to use single antiplatelet therapy rather than dual antiplatelet therapy (DAPT). A follow-up MRI revealed no significant enlargement in the extent of the acute-phase cortical infarction, indicating no progression of the infarction into the subcortical or deep white matter on DWI (Figure 2A). However, there was evidence of worsening cytotoxic and vasogenic edema in the cortical infarction regions on FLAIR imaging (Figure 2B). TOF revealed unchanged right MCA occlusion and persistent left MCA stenosis (Figure 2C). PWI using Tmax maps showed no significant changes in perfusion deficits (Figure 2D).

**Figure 3:**
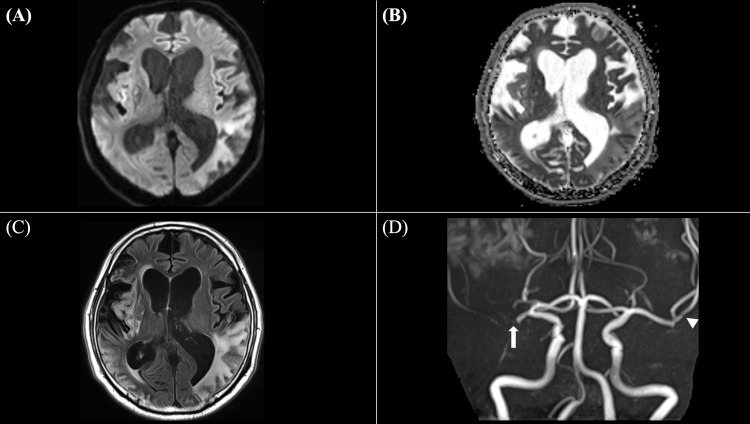
Follow-up MRI findings on hospital day 21 A: diffusion-weighted imaging (DWI) reveals no new hyperintense lesions, confirming the absence of new ischemic events and indicating stable infarct evolution; B: apparent diffusion coefficient (ADC) map shows no newly decreased ADC values, while previously restricted areas demonstrate ADC elevation, indicating subacute infarct evolution; C: fluid-attenuated inversion recovery (FLAIR) imaging demonstrates persistent vasogenic edema in the cortical infarction areas, without new signal abnormalities; D: time-of-flight (TOF) magnetic resonance angiography (MRA) shows persistent right middle cerebral artery (MCA) occlusion (arrow) and persistent left MCA stenosis (arrowhead), with no improvement of vascular patency.

Transthoracic echocardiography and carotid ultrasonography revealed no significant abnormalities, including the absence of valvular disease, intracardiac thrombus, or carotid artery stenosis. Lower limb venous ultrasonography showed no evidence of deep vein thrombosis. Continuous cardiac monitoring showed no arrhythmias, including atrial fibrillation. His level of consciousness gradually improved, but cognitive impairment and left-dominant quadriplegia persisted. On hospital day 21, a follow-up MRI revealed no new infarcted area (Figure 3A-3C) and unchanged right MCA occlusion and left MCA stenosis (Figure 3D).

On hospital day 90, he was transferred to a long-term care hospital with a modified Rankin Scale score of 4. At the time of transfer, MMT scores showed improvement: right upper limb, 5/5; left upper limb, 3/5; right lower limb, 5/5; left lower limb, 2/5, indicating partial recovery of motor function.

## Discussion

Cerebral infarction following anaphylaxis is a rare but clinically significant complication that presents diagnostic and therapeutic challenges due to its complex pathophysiology and the lack of established treatment guidelines [6]. The underlying mechanisms of anaphylaxis-associated cerebral infarction are multifactorial and may involve systemic hypotension and cerebral hypoperfusion, direct vascular dysfunction resembling Kounis syndrome, and a hypercoagulable state triggered by inflammatory and hemodynamic alterations. Understanding these mechanisms is essential for timely recognition and appropriate management.

Kounis syndrome is a hypersensitivity-induced vascular disorder that is traditionally recognized as an acute coronary syndrome caused by an allergic reaction [8]. However, the same pathophysiological mechanisms that underlie Kounis syndrome in the coronary arteries, such as mast cell degranulation, endothelial dysfunction, arterial vasospasm, and plaque destabilization, may also affect the cerebral vasculature, leading to ischemic stroke [4]. Kounis syndrome is classified into three types based on arterial status: Type I occurs in patients with normal arteries and is characterized by arterial spasm due to the acute release of inflammatory mediators; Type II affects patients with pre-existing atheromatous disease, in which the allergic reaction may precipitate plaque rupture; and Type III is observed in patients with arterial stent thrombosis [5]. In our case, MRI findings were consistent with ischemic changes localized to the cortical branches of the MCA, with infarcts restricted to the gray matter without extension into the subcortical or deep white matter. This distribution differs from the scattered, multifocal infarcts typically seen in cardiogenic embolic strokes, which frequently involve both cortical and subcortical regions [9]. Follow-up MRA performed three weeks later revealed persistent MCA occlusion and stenosis, suggesting the presence of pre-existing chronic vascular pathology rather than transient vasospasm. These findings indicate that our case most closely aligns with Type II Kounis syndrome, which is characterized by allergic inflammation leading to endothelial dysfunction, platelet activation, and plaque instability. In this scenario, the allergic reaction likely exacerbated pre-existing atheromatous disease, resulting in a critical reduction in cerebral perfusion and subsequent infarction. Thus, these findings highlight the importance of early hemodynamic stabilization in anaphylactic shock, as prolonged hypotension and systemic inflammatory responses can exacerbate ischemic injury, particularly in patients with pre-existing cerebrovascular disease.

Anaphylaxis also disrupts coagulation pathways, creating a hypercoagulable state that predisposes patients to thrombotic events. In our patient, laboratory findings demonstrated an elevated hematocrit of 58.6%, a red blood cell count of 5.96×106/μL, a hemoglobin level of 19.0 g/dL, and a D-dimer of 55.5 μg/mL. These hematologic abnormalities suggest hemoconcentration, which is likely a consequence of fluid extravasation due to increased vascular permeability caused by mast cell degranulation. During anaphylaxis, mast cell-derived mediators, such as histamine and platelet-activating factor, induce endothelial dysfunction and capillary leakage, leading to a shift of intravascular fluid into the interstitial space [2,3]. This results in a relative increase in hematocrit and blood viscosity, further compromising cerebral perfusion and exacerbating the risk of thrombosis [1,3,6]. In addition, pre-existing vascular risk factors, such as diabetes and hypertension, likely amplified this patient’s susceptibility to ischemic complications. These underlying conditions may have acted synergistically with anaphylaxis-induced hypoperfusion, hemoconcentration, and coagulation abnormalities, ultimately leading to cerebral infarction.

The management of cerebral infarction following anaphylaxis requires a comprehensive approach that addresses both the allergic reaction and the ischemic event. In our case, after neuroimaging confirmed the absence of hemorrhagic transformation, antiplatelet therapy with aspirin was initiated as part of secondary stroke prevention. The decision to use single antiplatelet therapy rather than DAPT was based on the extensive infarction and the high risk of hemorrhagic transformation associated with coagulopathy in anaphylaxis [10]. In cases of severe ischemia, the potential role of reperfusion therapy, such as tissue plasminogen activator or endovascular therapy, must be carefully considered [11]. While reperfusion therapy is the standard of care for acute ischemic stroke, its application in anaphylaxis-associated cerebral infarction is complicated by systemic hypotension, coagulopathy, and potential vascular fragility resulting from the allergic reaction [12]. In our case, reperfusion therapy was not selected because neuroimaging findings suggested the absence of salvageable ischemic tissue. DWI-FLAIR mismatch was not observed, indicating that the infarcted regions had already progressed beyond the hyperacute phase [13]. Additionally, Tmax maps showed prolonged transit time in the infarcted regions, but these areas closely corresponded to the DWI lesions, suggesting that there was no significant perfusion-diffusion mismatch indicative of salvageable penumbra [14]. Together, these findings confirmed that the affected brain tissue was already irreversibly damaged, making reperfusion therapy unlikely to provide clinical benefit [13,14]. Despite aggressive supportive care and rehabilitation, the patient remained functionally dependent at discharge, with a modified Rankin Scale score of 4. Given the rarity of this condition, long-term outcomes remain unclear, and further studies are needed to investigate optimal rehabilitation strategies and secondary prevention measures. Overall, this case highlights the critical importance of a multidisciplinary approach, including emergency physicians, neurologists, and radiologists, in managing anaphylaxis-related complications. Timely neuroimaging and comprehensive evaluation are essential to distinguish between vasospasm, embolic events, and other causes of cerebral infarction, ultimately guiding tailored and effective treatment strategies.

## Conclusions

Cerebral infarction following anaphylaxis, though rare, represents a potentially devastating complication, particularly in patients with pre-existing vascular risk factors. Our case illustrates that anaphylaxis-induced hypoperfusion, endothelial dysfunction, and hypercoagulability can act synergistically to precipitate ischemic stroke. Distinguishing anaphylaxis-associated stroke from other ischemic stroke etiologies is critical for guiding appropriate management strategies. Early recognition of neurological deficits, prompt neuroimaging, and rapid hemodynamic stabilization are therefore essential to prevent irreversible ischemic damage. These findings highlight the necessity of a coordinated, multidisciplinary approach for optimal management of anaphylaxis-related cerebral infarction. Further research is needed to better understand the pathophysiology and long-term outcomes, as well as to develop tailored therapeutic approaches for this rare but serious condition.

## References

[1] Simons FE, Ebisawa M, Sanchez-Borges M (2015). 2015 update of the evidence base: World Allergy Organization anaphylaxis guidelines. World Allergy Organ J.

[2] Reber LL, Hernandez JD, Galli SJ (2017). The pathophysiology of anaphylaxis. J Allergy Clin Immunol.

[3] Peavy RD, Metcalfe DD (2008). Understanding the mechanisms of anaphylaxis. Curr Opin Allergy Clin Immunol.

[4] Soufras GD, Kounis GN, Kounis NG (2014). Brain injury due to anaphylactic shock: broadening manifestations of Kounis syndrome. Int Endod J.

[5] Abdelghany M, Subedi R, Shah S, Kozman H (2017). Kounis syndrome: a review article on epidemiology, diagnostic findings, management and complications of allergic acute coronary syndrome. Int J Cardiol.

[6] Shankar T, Vempalli N, Asokan R, Pillai A, Infimate DJ (2023). Stroke in a patient of anaphylaxis-a case report and brief review. Int J Emerg Med.

[7] Hidayat R, Mesiano T, Kurniawan M (2023). Anaphylactic reaction as an etiology of ischemic stroke: a case report. Radiol Case Rep.

[8] Kounis NG, Zavras GM (1991). Histamine-induced coronary artery spasm: the concept of allergic angina. Br J Clin Pract.

[9] Sharobeam A, Churilov L, Parsons M, Donnan GA, Davis SM, Yan B (2020). Patterns of infarction on MRI in patients with acute ischemic stroke and cardio-embolism: a systematic review and meta-analysis. Front Neurol.

[10] Bhatia K, Jain V, Aggarwal D (2021). Dual antiplatelet therapy versus aspirin in patients with stroke or transient ischemic attack: meta-analysis of randomized controlled trials. Stroke.

[11] Takeuchi S, Miyauchi M, Kadota T, Fukuda M, Nishiyama K (2023). Cerebral infarction after anaphylactic shock due to cold-induced urticaria. QJM.

[12] Kounis NG, Koniari I, Velissaris D, Tzanis G, Hahalis G (2019). Kounis syndrome—not a single-organ arterial disorder but a multisystem and multidisciplinary disease. Balkan Med J.

[13] Thomalla G, Cheng B, Ebinger M (2011). DWI-FLAIR mismatch for the identification of patients with acute ischaemic stroke within 4·5 h of symptom onset (PRE-FLAIR): a multicentre observational study. Lancet Neurol.

[14] Kakuda W, Lansberg MG, Thijs VN (2008). Optimal definition for PWI/DWI mismatch in acute ischemic stroke patients. J Cereb Blood Flow Metab.

